# Long-term monitoring data logs of a recirculating artificial seawater based colonial ascidian aquaculture

**DOI:** 10.1016/j.dib.2021.107372

**Published:** 2021-09-15

**Authors:** Marta K. Wawrzyniak, Lluìs Albert Matas Serrato, Simon Blanchoud

**Affiliations:** Department of Biology, University of Fribourg, Chemin du Musée 10, 1700 Fribourg, Switzerland

**Keywords:** Colonial ascidians, Recirculating artificial seawater, In-land aquaculture, Water quality, Breeding monitoring, Marine model organism, Microbiota, *Botrylloides diegensis*, *Botryllus schlosseri*

## Abstract

This article presents and describes data related to the monitoring of our in-land in-lab marine recirculating artificial seawater husbandry system for breeding colonial ascidians [1] over a timespan of three years. These datasets were collected both automatically as well as manually, and include abiotic parameters (salinity, pH, temperature, ORP), concentrations of noxious ions (NH_4_^+^, NO_2_^−^, NO_3_^−^, PO_4_^3−^), the full lineaging of the colonies developing in the aquaculture setup, animal countings under four different feeding diets and animal survival in artificial seawater containing six different microbiota. Our aquaculture was used to breed two species of model colonial ascidians, *Botrylloides diegensis*[2], [3] and *Botryllus schlosseri*[4]. All the datasets are provided as raw CSV files together with an analysis script to reproduce the figures of our accompanying research article [1]. These extensive datasets give detailed insights into the impact of culturing conditions on the breeding of colonial ascidians and could be used to investigate this intricate relationship.

## Specifications Table

**Table:** 

Subject	Marine Biology
Specific subject area	In-land in-lab breeding of colonial ascidians in recirculating artificial seawater
Type of data	Table
How data were acquired	The data was acquired using an Apex controller (Neptune Systems, US) with five temperature sensors, two salinity sensors, one pH sensor and one ORP sensor; manually using water quality kits (Tropic Marin, DE) for pH, NH_4_^+^, NO_2_^−^, NO_3_^−^, PO_4_^3−^ and a refractometer (Red Sea, US) for salinity; and manually by visual inspection when handling the colonies for the lineages, the differential feeding and the microbiota experiments.
Data format	Raw (CSV text file)
Parameters for data collection	All available sensor readings were included in the dataset as well as all manual measurements over a monitoring duration of 3 years. Water quality parameters were gradually included in the dataset over the duration of the monitoring.
Description of data collection	Sensor readings were acquired automatically ever 200 s, water quality and feeding data were measured weekly, lineaging was acquired whenever colonies were manipulated, microbiota data was acquired daily for five consecutive days and on day 7.
Data source location	Institution: Department of Biology, University of Fribourg City/Town/Region: Fribourg/Fribourg Country: Switzerland Latitude and longitude (and GPS coordinates, if possible) for collected samples/data: 46.7932731,7.1556777
Data accessibility	With the article
Related research article	M.K. Wawrzyniak, L.A. Matas Serrato, S. Blanchoud, Artificial seawater based long-term culture of colonial ascidians, Developmental Biology. 480, pp. 91–104, https://doi.org/10.1016/j.ydbio.2021.08.005.

## Value of the Data

• These data provide quantitative information on breeding colonial ascidians under controlled environmental conditions at an unprecedented high resolution for a very long duration.
• These data are useful for researchers studying breeding of colonial ascidians and for groups interested in establishing colonial ascidians as in-lab model organisms.
• These data can be used in future experiments to investigate the impact of culturing conditions on breeding of colonial ascidians and as a basis for determining further optimized breeding conditions.

## 1 Data Description

This dataset is composed of 13 CSV files (comma-separated values, a standard flat text file format) containing the raw data of our monitoring. We here detail the content of each of these files.

Water_quality.csv contains the values from our water quality monitoring measurements ranging from April 20^th^ 2018 to February 12^th^ 2021. The file has one header line and 7 columns (Date, NH_4_^+^, NO_2_^−^, NO_3_^−^, Salinity, pH, PO_4_^3−^). Date format is DD/MM/YYY. Missing values are indicated by an empty cell.

Apex_ORP_Sump.csv contains the sensor readings of the Apex ORP (oxidation reduction potential probe located in the sump of our husbandry system [1], ranging from January 3^rd^ 2019 to February 15^th^ 2021. The file has no header row, and 2 columns (Date, value). Date is in POSIX time format (number of seconds elapsed since January 1^st^ 1970) using local Swiss time (Central European Time +1, with daylight savings). Readings were logged every 200 s (3 min 20 s), with the exception of occasional shutdown periods (software crash, reboot, updates). ORP was measured in mV.

Apex_pH_Sump.csv, Apex_Salt_Sump.csv and Apex_Temp_Sump.csv contain the sensor readings for pH, salinity and temperature in the sump of the system,respectively. File format is identical to that of Apex_ORP_Sump.csv. Salinity was measured in g/L and temperature in °C.

Apex_Temp_Tank1.csv, Apex_Temp_Tank2.csv, Apex_Temp_Tank3.csv and Apex_Temp_Tank4.csv contain the temperature readings in each specimen tank [1], formatted as the other Apex files. Apex_Salt_Tank4.csv contains the salinity readings in specimen tank 4 using the same format as those taken in the sump.

Strains_lineages.csv contains the lineaging data for all the animals bred in our husbandry setup, ranging from March 4^th^ 2019 to February 12^th^ 2021. The file has one header row and 8 columns (Name, Slide ID, Clone of slide ID, Treatment, Size, Location, Species, Added on). Name is a unique colony ID using the format SXXXCYYY where XXX is the strain ID and YYY the clone ID, e.g. S032C001 is the first clone of strain 32. Slide ID is an integer value representing which numbered microscopy glass slide the colony is settled on, or an alphanumeric value for large glass slides. Clone of slide ID is an integer value representing which numbered glass slide is the parent of this clone, this value is empty for the first clone of each strain. Treatment is a free text column where all the information relevant to the lineaging of a colony is described, each entry separated by a semi-column and ordered chronologically with the most recent event first and the corresponding date typically user the format (DD/MM/YY). Size is a description of the current surface covered by the colony that can take three values (small < 64 mm^2^, medium < 227 mm^2^ or large). Location is the tank [1] the colony is currently culture in (T1: Specimen tank 1, T2, T3, T4, Q1: Quarantine tank 1, Q2). Species is the type of animal being culture (*Botrylloides* sp., *Botryllus schlosseri, Ciona*). Added on is the creation date of the colony ID using the format MM/DD/YYYY HH:MM. Missing values are indicated by an empty cell.

Differential_feeding.csv contains the data on animal countings during a differential feeding experiment spanning between September 11^th^ 2020 and November 13^th^ 2020. The file has one header row and 4 columns (Food mix, slide ID, date, No adults). Food mix is a one character identifier specifying the feeding diet. Slide ID is an integer value representing which numbered microscopy glass slide the counted colony is settled on. Date is the date of the counting using the format DD.MM.YY. No adults is the number of adults present in the colony.

Microbiota_survival.csv contains the data on animal countings during the microbiota survival experiments. Each experiment lasted one week. The file has one header row and 7 columns (ImageStack, Slice, Experiment, Dpi, SlideID, Side, Count). ImageStack is an integer value indicating in which original picture stack the data was obtained from, with Slice specifying at which position inside that stack. Experiment is an integer value indicating which experiment was being measured, with 0 being no inoculation, 10 standard system water and other values the corresponding bacteria strain. Dpi provides the number of days post inoculation of when the data was acquired. SlideID is an integer value representing which numbered microscopy glass slide the counted colony is settled on. Side is an integer value indicated which side of the slide was counted, with 1 being the front, 2 the back and 3 both sides. Count is the number of adults present on the specified side of the colony.

## 2 Experimental Design, Materials and Methods

Data collection was designed to closely monitor the abiotic parameters that could influence the water quality of our recirculating husbandry system together with the propagation of the colonial ascidians bred in it. Monitoring was undertaken to promptly identify deviations of the culturing environment in our system to correct them for providing a stable breeding system comparable to the natural environment of our animals [5].

Salinity, pH, temperature and ORP were measured automatically every 200 s (3 min 20 s) and stored by an Apex controller (Neptune Systems, US). Double-junction lab grade sensors for salinity, pH, temperature and ORP were placed in the sump of our husbandry system, close to the tubing delivering brine water, held using a magnetic stand sufficiently deep in the tank to ensure constant submersion and their double junction kept dry using a tight silicone tube isolating the rear of the sensors from the water [1]. Four additional temperature probes monitored the temperature in each specimen tank, and a second salinity sensor monitored tank 4 for redundancy. The additional sensors in tanks 1 and 3 were connected to the controller through two pH/ORP/Redox modules, while those for tanks 2 and 4 through two Salinity modules. All sensors were sampled simultaneously. All sensors were calibrated by Neptune Systems.

Concentrations of noxious ions (NH_4_^+^, NO_2_^−^, NO_3_^−^, PO_4_^3−^) were measured manually weekly using colorimetric kits (Tropic Marin, DE) together with pH, also using a Tropic Marin colorimetric kit, and salinity using a refractometer (Red Sea, US). 100 mL of system water was collected from the outlet of the refugium within the sump of our husbandry system and left 10 min to temper on the bench. Colorimetric kits were used according to the manufacturer's instructions. Refractometer was calibrated following the manufacturer's instructions using deionized water. Proper calibration was verified before every measure. PO_4_^3−^ measurements were included starting from November 6^th^ 2019 after all the ions measurable using Tropic Marin colorimetric kits were tested and it was the only one outside the recommended range.

Salinity and pH probes were re-calibrated using commercially available solutions when discrepancies were observed either between the probes or with the manual measurements. Re-calibration was done twice for salinity and once for pH over the 3 years of the monitoring.

Colony monitoring was performed as required by the animals but at least twice per month. Animals were cleaned using a fine paint brush and dirty or unhealthy tissue ablated using a single-edge razor blade. All treatments were manually recorded in the corresponding dataset. Creation of new clones was typically done by subcloning a parent colony [6], and entered in the dataset on the same day as the surgery such that the creation date of the data entry corresponds to the creation date of the clone. When updating the dataset could not be done on the same day, a mention stating the subcloning date was added to the treatments entry. Additional treatments recorded include positioned, re-positioned, dead, sacrificed, removed, fixed, missing and slipped. Data entries related to dead clones were modified by using the special Slide ID 0 and by removing their Size and Location information.

Differential feeding experiment was designed using 4 sets of three clones, all sets being composed by the same three strains. Each tank was fed with a given diet for 4 consecutive weeks, the colonies from tanks 1 and 3, as well as 2 and 4, were swapped and the tanks fed for another 4 weeks. All 12 colonies were monitored weekly to manually count the number of adults that composed the clone.

Microbiota survival experiment was designed using 10 sets of three clones, all clones belonging to the same strain (S131). Each microbiota was tested separately for its impact on the survival of our colonies over the span of one week. A 10 L tank was filled with 7 L of artificial seawater inoculated with 50 µL of the corresponding bacterial strain, or with water from our recirculating system. Colonies were fed once a day with 20 µL of our food mix [1] and monitored daily for the first 4 days, as well as a final time after 7 days, to manually count the number of adults that composed the colony. parse_tunicate_husbandry.m is a GNU Octave script that parses each file of the presented dataset to produce all the quantitative panels of in our accompanying article [1].

## Ethics Statement

All experiments comply with the ARRIVE guidelines and were carried out in accordance with the Swiss Animal Welfare Ordinance, 2008 and the Swiss Animal Protection Act, 2005.

## CRediT authorship contribution statement

**Marta K. Wawrzyniak:** Investigation, Data curation, Validation, Writing – review & editing. **Lluìs Albert Matas Serrato:** Investigation, Data curation, Validation, Writing – review & editing. **Simon Blanchoud:** Conceptualization, Funding acquisition, Project administration, Supervision, Writing – original draft.

## Declaration of Competing Interest

The authors declare that they have no known competing financial interests or personal relationships which have or could be perceived to have influenced the work reported in this article.

## Appendix Supplementary materials

Image, application 1
